# Cardiometabolic risk factors and quality of life in severely obese children and adolescents in the Netherlands

**DOI:** 10.1186/1471-2431-13-62

**Published:** 2013-04-22

**Authors:** Sabine Makkes, Carry M Renders, Judith E Bosmans, Olga H van der Baan-Slootweg, Jacob C Seidell

**Affiliations:** 1 Department of Health Sciences and the EMGO Institute for Health and Care Research, VU University Amsterdam, Amsterdam, The Netherlands; 2 Merem Treatment Centers, Heideheuvel, Hilversum, The Netherlands

**Keywords:** Severe obesity, Child, Adolescent, Cardiometabolic risk factors, Quality of life

## Abstract

**Background:**

The prevalence of severe obesity in children and adolescents is increasing. However, little is known about cardiometabolic risk factors and quality of life of children with severe obesity.

Therefore, the aim of this study was to assess the demographic characteristics and the prevalence of cardiometabolic risk factors and quality of life in severely obese children and adolescents undergoing intensive inpatient treatment for obesity.

**Methods:**

Data were collected between August 2009 and April 2011 on 16 children (8-13y) and 64 adolescents (13-19y) with severe obesity (SDS-BMI >= 3.0 or SDS-BMI >= 2.3 and comorbidity) participating in an RCT evaluating two intensive inpatient treatment programs for obesity. Demographic, anthropometric, clinical characteristics and two components of the EuroQol for the assessment of quality of life are described.

**Results:**

Eighty percent of participants in this study had at least one cardiometabolic risk factor in addition to severe obesity. Low HDL-cholesterol and hypertension were most prevalent (65.0% respectively 31.2%). The highest significant correlations were found between SDS-BMI and SDS-waist circumference, fasting plasma insulin and HOMA-IR (correlation coefficients respectively 0.80, 0.49, and 0.48). With regard to quality of life, the mean utility score of the participants was 0.79 on a scale of 0.0 to 1.0 on the EuroQol questionnaire and their mean individual valuation was 69.1 on a scale of 0 to100.

**Conclusion:**

Cardiometabolic risk factors are already highly prevalent in this group of severely obese children and adolescents. The score of 69.1 found for quality of life in this study suggests that participants experience important limitations in their quality of life. However, quality of life is not associated with the prevalence of cardiometabolic risk factors.

**Trial registration:**

Netherlands Trial Register (NTR1678, registered 20-Feb-2009)

## Background

Worldwide there has been a large increase in the prevalence of obesity in children and adolescents in the last decades [1,2]. In 2009 in the Netherlands, about 2% of the boys and girls were classified as obese. In comparison with 1980 these figures indicate a four to six fold higher prevalence for obesity [3]. Moreover, the severity of obesity has also increased, both in the Netherlands as in other countries. Results from the 2009–2010 NHANES indicate that an estimated 16.9% of children and adolescents aged 2–19 years in the US are obese [4]. The rate of severe childhood obesity has tripled in the last 25 years [5]. This trend is worrisome, because obese children have a higher risk of type 2 diabetes mellitus and cardiometabolic risk factors such as high blood pressure, low HDL cholesterol, high triglycerides and high fasting insulin concentration [6-9]. These cardiometabolic risk factors are often clustered together in individuals which increases the risk of cardiometabolic disease in young adulthood [10]. In addition, childhood obesity often tracks into adulthood and is related to cardiometabolic disease, diabetes type 2 and even cancer in later life, independent of adult BMI [11-13]. Besides these negative clinical consequences of obesity, low self esteem and behavioral problems seem to be particularly common in obese children [10]. Obese children and adolescents also have psychological problems and a lower health-related quality of life [14]. Although it can be expected that the health consequences of severe obesity are even more serious than of obesity, little is known about the demographic, anthropometric and clinical characteristics of severely obese children and adolescents. This information is important as it will give more insight into the health risks of these children. Therefore, the objective of this article is to assess the prevalence of cardiometabolic risk factors and the quality of life in severely obese children and adolescents undergoing intensive inpatient treatment for obesity in the Netherlands.

## Methods

### Design

Data were collected between August 2009 and April 2011 on 16 children (8-13y) and 64 adolescents (13-19y) with severe obesity who participated in the Health Effects of Lifestyle Interventions in severely Obese children and adolescents Study (HELIOS). HELIOS is a randomized controlled trial (RCT) in which the cost-effectiveness of two treatment programs aimed at changing the lifestyle of both participants and their parents is compared. These children receive one year of intensive treatment starting with either an inpatient period of two or six months in a tertiary obesity center in Hilversum, the Netherlands. After the intensive treatment period, there is follow-up of one year. Details about the design of the study and the two treatment programs can be found elsewhere [15].

### Participants

Inclusion criteria for the trial were a SDS-BMI >= 3.0 (this corresponds to the 99.9th percentile) according to the growth curves based on the fourth Dutch nationwide growth study of 1997, or a SDS-BMI >= 2.3 (this corresponds to the 99th percentile) and comorbidity (e.g. obstructive sleep apnea syndrome, raised insulin, diabetes type II, liver function disorders, dyslipidemia, worn out joints). Participants were excluded from the trial if they had syndromal or chromosomal determined obesity; obesity caused by endocrine disorders (hypothyroidism, Cushing syndrome, primary hyperinsulinemia, pseudohypoparathyroidism, acquired (structural) hypothalamic damage) or medicine use (e.g. oral steroids, antiepileptic drugs, antidepressants); psychiatric problems; an IQ below 75 or similar school level or if their parents were not willing or able to participate in the treatment program and/or study.

### Measurements

Data on demographic characteristics, comorbidities and quality of life were obtained by questionnaires completed by the participants and their parents. Participants were categorized into two main groups for ethnicity; Native Dutch and Immigrant (Western and Non-Western). When both parents were born abroad in different countries, the country in which the mother was born was used to classify the participant [16]. Highest educational level attained by one of the parents was used for analysis and is divided into low (lower vocational training, lower general secondary education and primary school and special primary education or less), medium (intermediate vocational training, higher general secondary training and pre-university education) or high (completed higher vocational training and university) [17,18]. To determine socio economic status (SES) of the participants, we used status scores of the parents using data from The Netherlands Institute for Social Research [19]. A status score is a measure for the social status of a postal code area and consists of three elements: income, level of education and level of unemployment. A status score below 0 means a SES above average and a status score above 0 means a SES below average (0 meaning average) [19]. Quality of life was measured using the EuroQol (EQ-5D-3 L) [20]. The EuroQol consists of two components; the EQ-5D descriptive system and the EQ visual analogue scale (EQ VAS). Both the EQ-5D and EQ-VAS were completed by the participants. The EQ-5D descriptive system consists of five dimensions (mobility, self care, usual activities, pain/discomfort, and anxiety/depression) with three levels of severity (no problems/some or moderate problems/extreme problems). The participants were asked to choose the level that best described their current health status for each dimension. The resulting health state was converted to a utility score using the Dutch EQ-5D valuation tariff [21]. Utilities represent quality of life in a single number along a continuum ranging from 0.0 (death) to 1.0 (full health). In the EQ-VAS a standard vertical 20 cm visual analogue scale is used to measure an individual’s direct valuation of their current health-related quality of life on a scale of 0 (worst imaginable health state) to 100 (best imaginable health state) [20]. Comorbidity (acanthosis nigricans, Blount’s disease, gallstones, hirsutism and pseudogynecomastia) was determined by the treating pediatricians from obesity center Heideheuvel before start of the treatment and by ultrasound. A clear description of the anthropometric measurements that were performed can be found elsewhere [15]. The equation used for the children and adolescents in this study to calculate fat mass percentage was the Kushner equation for total body water (TBW) adjusted by Newton et al. [22,23]. After an overnight fast, blood samples were obtained to measure lipid spectrum, high sensitive C-reactive protein (HS-CRP) and hemoglobin type A1C (HbA1C). To determine glucose tolerance and insulin resistance, the participants were given glucose, in a dose of 1.75 g per kilogram of body weight (up to a maximum of 75 g) orally. Blood samples were obtained at 0 and 120 minutes for the measurement of glucose and insulin levels. Homeostasis model assessment for insulin resistance (HOMA-IR) was calculated using the following equation: fasting plasma insulin (μU/L) x fasting glucose (mmol/L)/22.5. Scores ordinarily range from 0 to 15, with higher scores indicating greater insulin resistance. Insulin resistance was determined using the cut-off points of Kurtoğlu et al. for participants in the prepubertal and pubertal stage (prepubertal: 2.67 in boys and 2.22 in girls, pubertal: 5.22 in boys and 3.82 in girls) [24,25]. For participants in the postpubertal stage, the adult cut-off point of > 2.5 from Keskin et al. was used [26]. The three pubertal stages, prepubertal, pubertal and postpubertal, were based on Tanner stages: prepubertal equals G/M1&P1; postpubertal equals G/M5&P5; pubertal equals all other Tanner stages. For all participants the presence of a number of well known cardiometabolic risk factors was determined [27,28]. The definitions of these cardiometabolic risk factors are included in the Additional file 1.

### Statistical analyses

All analyses were performed with IBM SPSS Statistics for Windows, Version 20. Results were stratified by age, where participants in the age group 8 to 13 years were categorized as ‘children’ and participants in the age group 13 to 19 years were categorized as ‘adolescents’. Results were also stratified according to SDS-BMI median (SDS-BMI = 3.49). Independent Student’s t-tests were used to analyze differences in continuous measures between the age groups. Chi-square tests were used to analyze differences in categorical variables. Because several variables had distributions that deviated from normality, Spearman’s rank correlations were calculated. Spearman’s rank correlations were used to explore the relationship between SDS-BMI and anthropometric and laboratory measurements, such as blood pressure, blood lipids, insulin and glucose. In addition to SDS-BMI, also the relationship between utility score and these outcomes was explored. Also Pearson product–moment correlation coefficients were calculated but results are not shown in the tables. In addition logistic regression was used to determine the association between SDS-BMI and dichotomous classification of elevated cardiometabolic risk factors. A p-value below 0.05 was considered statistically significant.

## Results

### Demographic characteristics

Table 1 describes the demographic characteristics of the 80 participants. Of the participants, the mean age of the group was 14.8 years, 66.2% were girls and 20% fell in the age group 8 to 13 years. Approximately half of the participants (55.0%) were native Dutch. Of the Immigrants, 5.0% were Western. Thirty-six percent of the participants’ parents were classified as having a low level of education and 40% and 17.5% as having a medium or high level of education, respectively. Over 60% of the participants lived in a family situation with a SES below average. About half of the participants lived in two parents family, 41.3% of the participants lived in a single parent family. No statistically significant differences were found in demographic characteristics between the children and adolescent groups, except for mean age. Acanthosis nigricans was the most common comorbidity among the participants (60%). Blount’s disease was only present in adolescents. The percentage of participants having gallstones differed significantly between children and adolescents, although in absolute numbers there were only 2 cases among children and 1 case among adolescents. Almost all boys had pseudogynecomastia, among children even 100%. Hirsutism was seen in approximately 9% of the girls. According to self reported data by the parents of the participants, the weight of the participants started to increase disproportionally when they were about 5 years old and at the age of 9 the weight started to become a real problem.

**Table 1:** Demographic characteristics, quality of life and comorbidity of the children and adolescents participating in HELIOS, for all participants together and stratified according to age group

	**Total (n = 80)**	**Children (n = 16)**	**Adolescents (n = 64)**
N	80	16	64
Age (y)	14.8 (2.3)	11.3 (1.2)*	15.7 (1.6)*
Male/Female (%)	33.8/66.2	50/50	29.7/70.3
Ethnicities (%)			
Native Dutch	55.0	68.8	51.6
Immigrants	45.0	31.2	45.5
o Western (% of total)	5.0	0.0	6.3
o Non-Western (% of total)			
▪ Moroccan	5.0	6.3	4.7
▪ Dutch Antilles/Aruba	5.0	0.0	6.3
▪ Surinam	5.0	0.0	6.3
▪ Turkish	16.3	18.8	15.6
▪ Other Non-Western	6.3	6.3	6.3
Level of education of the parents (%)			
Low	36.3	62.5†	29.7†
Medium	40.0	25.0†	43.8†
High	17.5	6.2†	20.3†
SES (%)			
Below average	62.5	73.3	63.9
Above average	32.5	26.7	36.1
Household situation (%)			
Married/living together	55.0	56.2	54.7
Divorced	33.8	37.5	32.8
One parent family(mother)	7.5	0.0	9.4
Other situation	3.8	6.2	3.1
Quality of life			
EQ-5D utility score	0.79 (0.22)	0.72 (0.27)	0.80 (0.20)
EQ VAS	69.1 (21.2)	76.1 (21.3)	67.4 (21.0)
Comorbidity (%)			
Acanthosis nigricans	60.0	56.2	60.9
Blount’s disease	3.8	0.0	4.7
Gallstones	3.8	12.5*	1.6*
Hirsutism (only girls)	9.4	12.5	9.1
Pseudogynecomastia (only boys)	92.6	100.0	89.5

Data are mean (SD).

Percentages are actual percentages, not valid percentages.

SD – standard deviation; SES – Socio-economic status.

A participant falls in the “Children” group if aged 8 to 13 years and falls in the “Adolescents” group if aged 13 to 19 years.

If both parents were born in the Netherlands, a participant was categorized as Native Dutch; if one of the parents was born abroad, a participant was categorized as Immigrant. Immigrant is further divided into Western of Non-Western, of which Non-Western if further subdivided into Morocco, former Dutch Antilles and Aruba, Surinam, Turkey and Other Non-Western.

SES below average corresponds to a status score of 0 or higher, SES above average corresponds to a status score below 0.

* P value <0.05.

† P value <0.10.

### Quality of life

**Figure 1:**
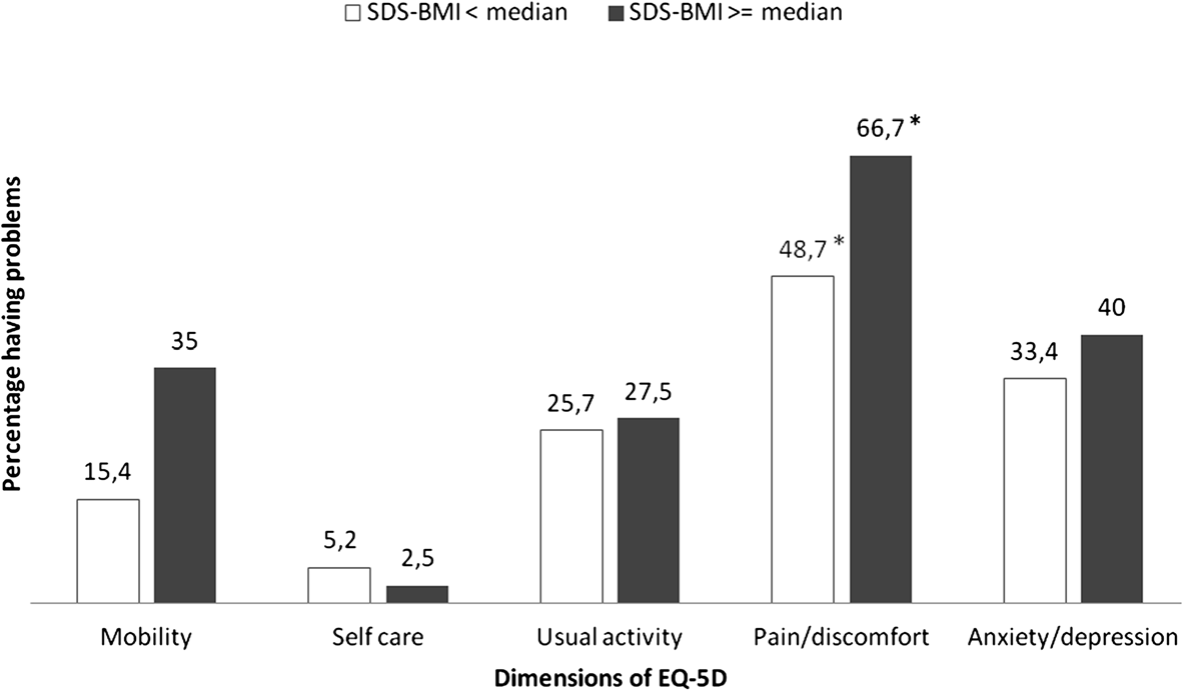
**Frequency of reported problems within the dimensions of the EQ-5D descriptive system stratified according to the median of SDS-BMI.** The EQ-5D descriptive system consists of five dimensions (mobility, self care, usual activities, pain/discomfort, and anxiety/depression). SDS-BMI – standard deviation of body mass index. Median is 3.49. * P value <0.05.

The mean EQ-5D utility score of the participants was 0.79 on a scale of 0.0 to 1.0 and their mean EQ VAS was 69.1 on a scale of 0 to 100. Children scored slightly lower than adolescents (0.72 vs. 0.80) on the EQ-5D. Participants with a SDS-BMI >= median had lower utility scores than participants with a SDS-BMI < median (0.77 vs. 0.80), although this was not significantly different. 20 children and adolescents (25%) reported having no problems at all on any of the five dimensions. Of the participants reporting any problems (75%), the most reported problem was pain; 57.3% reported having (some or extreme) problems with pain. This was followed by anxiety/depression (36.3%), usual activity (26.3%), mobility (25.1%) and finally self care (3.8%). No differences were found between the adolescents and children. However, participants with a SDS-BMI >= median had slightly more problems with mobility (35% vs. 15.4%) and pain (66.7% vs. 48.7%) than participants with a SDS-BMI < median, although for mobility this was not statistically significant (Figure 1). Of the participants reporting any problems (some or extreme) on any of the five dimensions, the majority experienced some problems. Extreme problems were most often reported on the anxiety/depression dimension. Relatively boys had slightly more problems with activity whereas girls tented to have more problems with anxiety/depression, although these differences were not statistically significant. As for the EQ-VAS, children had a higher EQ VAS than adolescents (76.1 vs. 67.4). Also participants with a SDS-BMI < median scored higher on the EQ VAS than participants with a SDS-BMI >= median (73.7 vs. 64.7). These differences were not statistically significant.

### Anthropometric measurements

Of the 80 participants, the mean SDS-BMI was 3.4 and the mean fat mass percentage was 52% (Table 2). The SDS-BMI, SDS-waist circumference, fat mass percentage, systolic blood pressure, fasting plasma insulin, fasting plasma glucose, triglycerides and HOMA-IR were significantly higher in participants with a SDS-BMI >= median than in participants with a SDS-BMI < median. Also the distribution of sexes differed significantly between the SDS-BMI groups, with more girls in the lower SDS-BMI group. There was a statistically significant difference in SDS-BMI and SDS-waist circumference between boys and girls; 3.7 vs. 3.3 and 3.8 vs. 3.5. With regard to age, adolescents had a statistically significant higher systolic blood pressure and HS-CRP than children (data not shown). SDS-BMI was significantly correlated with SDS-waist circumference, fat mass percentage, systolic blood pressure, fasting plasma insulin, triglycerides and HOMA-IR (correlation coefficients respectively 0.80, 0.32, 0.37, 0.49, 0.37 and 0.48). We also looked at correlations between EQ-5D utility scores and anthropometric and laboratory measurements, but no significant correlations were found. No relevant differences in anthropometric measurements were found between participants with an EQ-5D utility score < median and participants with a score >= median.

**Table 2:** Anthropometric and laboratory measurements of the children and adolescents participating in HELIOS, for all participants together and stratified according to SDS-BMI group and Spearman’s rank correlations

	**Total (n = 80)**	**SDS-BMI < median (n = 39)**	**SDS-BMI >= median (n = 41)**	**Spearman’s R with SDS-BMI**	**Spearman’s R with EQ-5D**
Age (y)	14.8 (2.3)	14.4 (2.4)	15.2 (2.2)	0.19	0.05
Male/Female (%)	33.8/66.2	17.9/82.1**	48.8/51.2**	NA	NA
SDS-BMI	3.4 (0.4)	3.1 (0.2)**	3.7 (0.3)**	-	−0.03
SDS-waist circumference	3.6 (0.3)	3.4 (0.2)**	3.8 (0.3)**	0.80**	−0.07
BIS					
Fat mass (%)	52.3 (4.4)	50.5 (3.8)**	53.9 (4.4)**	0.32**	−0.13
Systolic blood pressure (mmHg)	121.9 (12.8)	117.5 (11.1)**	126.1 (13.0)**	0.37**	−0.09
Diastolic blood pressure (mmHg)	76.8 (11.1)	74.9 (9.4)	78.7 (12.4)	0.19	0.03
Puberty stage (%)^1^					
Prepubertal	8.8	12.8	4.9	NA	NA
Pubertal	48.8	53.8	43.9	NA	NA
Postpubertal	42.5	33.3	51.2	NA	NA
Fasting plasma insulin (pmol/L)	98.6 (65.1)	72.6 (40.4)**	122.7 (74.4)**	0.49**	−0.12
Fasting plasma glucose (mmol/L)	4.7 (0.3)	4.6 (0.3)*	4.8 (0.3)*	0.19	−0.10
2-h plasma glucose (mmol/L)	5.8 (1.2)	5.8 (1.4)	5.9 (0.9)	0.14	−0.22†
Total cholesterol (mmol/L)	3.8 (0.7)	3.7 (0.8)	3.8(0.7)	0.09	−0.13
HDL-cholesterol (mmol/L)	1.0 (0.2)	1.1 (0.2) †	1.0 (0.2) †	−0.22†	−0.10
LDL-cholesterol (mmol/L)	2.3 (0.6)	2.2 (0.6)	2.3 (0.7)	0.00	−0.08
Triglycerides (mmol/L)	1.0 (0.6)	0.8 (0.4)**	1.2 (0.6)**	0.37**	−0.18
HS-CRP (mg/l)	5.0 (4.6)	4.4 (4.6)	5.5 (4.6)	0.22	0.07
HbA1C (DCCT %)	5.5 (0.3)	5.5(0.3)	5.5 (0.2)	0.05	0.07
HOMA-IR	3.0 (2.1)	2.2 (1.3)**	3.8 (2.4)**	0.48**	−0.15

Data are mean (SD).

SD – standard deviation; SDS-BMI – standard deviation of body mass index; SDS-waist circumference – standard deviation of waist circumference; BIS – Bio-electrical impedance spectroscopy; HDL – high-density lipoprotein; LDL – low-density lipoprotein; HS-CRP - high sensitive C-reactive protein; HbA1C – hemoglobin type A1C; HOMA-IR – homeostasis model assessment for insulin resistance; EQ-5D – EQ-5D descriptive system; R – correlation.

Median is 3.46.

† P value = 0.05.

* P value <0.05.

** P value < 0.01.

^1^ Three stages based on Tanner stages: ‘prepubertal’ equals G/M1&P1; ‘postpubertal’ equals G/M5&P5; ‘pubertal’ equals all other Tanner stages.

NA Not applicable.

### Cardiometabolic risk factors

Table 3 shows that 80% of all participants had at least 1 cardiometabolic risk factor in addition to being obese. This was 74.4% in the lower SDS-BMI group and 85.4% in the higher SDS-BMI group. The most common cardiometabolic risk factors were low HDL-cholesterol (65.0%), hypertension (31.2%) and high triglycerides (11.2%). These risk factors were more present among participants with a SDS-BMI above the median than among participants with a SDS-BMI under the median, although these differences were not significant. Furthermore, children and adolescents with a higher SDS-BMI were 5.5 times more likely to have hypertension (OR 5.51, 95% CI 0.55, 22.18) and more than five times more likely to have a high insulin resistance (OR 5.35, 95% CI 1.39, 20.63). In the group with a SDS-BMI >= median fewer participants had no cardiometabolic risk factor in addition to their obesity than in the group with a SDS-BMI < median. Of the participants that had any cardiometabolic risk factor in addition to obesity, more children and adolescents in the highest SDS-BMI group had 2 or more cardiometabolic risk factor in addition to their obesity, whereas more children and adolescents in the lower SDS-BMI group had only 1 cardiometabolic risk factor in addition to their obesity. Additional analyses in which results were stratified by SDS-BMI < 3.0 (N = 9) or SDS-BMI >= 3.0 (N = 71) taken the inclusion criteria into account, showed that 66.7% respectively 81.7% had at least 1 cardiometabolic risk factor or more in addition to being obese (data not shown). When we looked at the group of children and the group of adolescents separately (data not shown), we saw that already 68.8% of the children between 8–13 years old had 1 cardiometabolic risk factor in addition to their obesity and 18.8% already had 2 additional cardiometabolic risk factors. Only 31.2% of the children had no additional cardiometabolic risk factors in addition to obesity, for adolescents this was even lower with 17.2% No differences were found for the different cardiometabolic risk factors between the group with a utility score < the median and the group with a utility score >= the median (data not shown). There was also no difference in prevalence of the number of risk factors between these groups. Participants with a higher utility score were not more likely to have any of the cardiometabolic risk factors included in Table 3 (data not shown). If we combine the cardiometabolic risk factors in our study according to the new IDF definition of MetS, the prevalence of MetS was 27.5% in our study population. When we took the degree of obesity into account, 23.1% in the lower SDS-BMI group had MetS, whereas 31.7% in the higher SDS-BMI group had MetS.

**Table 3:** The prevalence of cardiometabolic risk factors (CRF) of the children and adolescents participating in HELIOS, for all participants together and stratified according to SDS-BMI group

	**Total (n = 80)**	**SDS-BMI < median (n = 39)**	**SDS-BMI >= median (n = 41)**	**OR (95% CI)**
High triglycerides	9 (11.2)	3 (7.7)	6 (14.6)	2.47 (0.42, 14.64)
Low HDL-cholesterol	52 (65.0)	24 (61.5)	28 (68.3)	1.49 (0.55, 4.94)
Hypertension	25 (31.2)	9 (23.1)	16 (39.0)	5.51 (1.37, 22.18)*
Impaired fasting glucose	1 (1.2)	1 (2.6)	0 (0)	-
Impaired glucose tolerance	5 (6.2)	4 (10.3)	1 (2.4)	-
DMII	0 (0.0)	-	-	-
High HOMA-IR	30 (37.5)	9 (23.1)**	21 (51.2)**	5.35 (1.39, 20.63)*
1 CRF (only obesity)	16 (20.0)	10 (25.6)	6 (14.6)	NA
2 CRF (1 in addition to obesity)	39 (48.8)	18 (46.2)	21 (51.2)	NA
>= 3 CRFs (>= 2 in addition to obesity)	25 (31.3)	11 (28.2)	14 (34.1)	NA

Data are n (% of total) and OR with 95% CI.

OR – Odds Ratio; SDS-BMI – standard deviation of body mass index; CI – confidence interval; HDL – High Density Lipoprotein; HOMA-IR – homeostasis model assessment for insulin resistance: DMII – Diabetes Mellitus type II.

Reference cut off points cardiometabolic risk from ‘The metabolic syndrome in children and adolescents – an IDF consensus report’ by Zimmet et al. [27].

Reference cut off points HOMA-IR from ‘Insulin resistance in obese children and adolescents: HOMA-IR cut-off levels in the prepubertal and pubertal periods’ by Kurtoğlu et al. [25], ‘Homeostasis model assessment is more reliable than the fasting glucose/insulin ratio and quantitative insulin sensitivity check index for assessing insulin resistance among obese children and adolescents’ by Keskin et al. for postpubertal stage [26].

CRFs are based on the IDF criteria including obesity, high triglycerides, low HDL-cholesterol, hypertension, impaired fasting glucose, impaired glucose tolerance and DMII. Since all participants fulfilled the IDF criterion for obesity, none of the participants had zero CRF. Participants with 1 CRF only had obesity and none of the other CRFs. Participants with 2 CRFs had 1 CRF in addition to obesity. Participants with 3 or more CRFs had 2 or more CRFS in addition to obesity. HOMA-IR is not taken into account in the number of CRFs.

Median is 3.49.

- Insufficient sample size.

* P value <0.05.

** P value < 0.01.

NA Not applicable.

## Discussion

This study shows that 80% of the severely obese children and adolescents eligible for intensive inpatient treatment programs for severely obese had at least 1 cardiometabolic risk factor (in addition to obesity) indicating that they are at increased risk for severe health complications. Within this group, higher SDS-BMI was associated with a worse profile of several cardiometabolic risk factors. The mean EQ VAS of the participants was 69.1 on a scale of 0 to 100. Unfortunately, there are hardly studies that describe characteristics of this specific group of severely obese children and adolescents. Therefore there is little insight into the health risks of this group and the urgency to quickly detect this group and offer suitable treatment programs. Most studies included only obese participants and not severely obese or also obese participants, or had fewer participants than we did. Moreover often anthropometric and clinical characteristics of the study population are only described after treatment. In our study, we see a tendency; the higher the SDS-BMI and higher the degree of obesity, the higher the prevalence of MetS. This is also seen in a study by Weiss et al. [29], although the z-scores of BMI in our study population are much higher. Weiss et al. found that 38.7% of the participants with moderate obesity (defined as 2.0 < SDS-BMI = < 2.5) and 49.7% with severe obesity (SDS-BMI > 2.5) were diagnosed with MetS. Thus, the prevalence of cardiometabolic risk factors seems to increase with worsening obesity, even in the upper regions. The most common cardiometabolic risk factor seen in our study population is low HDL-cholesterol (65.0%), followed by hypertension (31.2%) and high triglycerides (11.2%). In the Lafortuna study, hypertension was the most prevalent component of MetS (66.1% for Germans vs. 44.7% for Italians), followed by low HDL-cholesterol (39.5% vs. 37.4% respectively). Adolescents had higher prevalences of cardiometabolic risk factors than children in our study population. This was also found by Lafortuna et al., in which the prevalences of almost all components of MetS increased with age groups. There are only a few studies that looked at the relationship between childhood obesity and quality of life, and almost none that studied this association in severely obese children and adolescents. In our study we found that the average score on a scale of 0 to 100 was 69.1. Since we only included severely obese children and adolescents in our study population, we cannot directly compare our results to a similar group of normal weight children. However, we did compare our results with existing literature describing quality of life in obese children and adolescents. The EQ-VAS score of 69 found in this study suggests that participants experience important limitations in their quality of life. This was also found by Schwimmer et al. who used the PedsQL to assess quality of life in obese and normal weight children [14]. They found that obese children had a significantly lower mean health-related quality of life score compared to healthy children and adolescents (mean score 67.0 vs. 83.0) and that they were 5.5 times more likely to have impaired health-related quality of life than a healthy child or adolescent, which is similar to a child or adolescent with cancer. Williams et al. also used the PedsQL 4.0 and compared schoolchildren with normal weight, overweight and obesity to each other [30]. They found total mean scores of respectively 80.5, 79.3 and 74.0. The degree of obesity was taken into account by Varni et al. [31]. Severely obese children self reported significantly lower overall health-related quality of life, physical health, psychosocial health, and school functioning in comparison to obese children. The results of a recent study by Philips et al. also suggest the ‘extremely obese’ are significantly more depressed, more socially anxious and have poorer quality of life in comparison to the ‘obese’ [32]. We report similar findings in our study (higher BMI, poorer quality of life), although our results were not statistically significant. We did not find a relationship between quality of life and the presence of cardiovascular risk factors, like Nadeau et al. did [33]. In their study, a higher body mass index and greater number of comorbidities were associated with diminished health-related quality of life. The difference may be explained by the fact that Nadeau et al. used a disease specific questionnaire, which is probably more sensitive to detect limitations related to obesity than the general quality of life questionnaire we used in our study. All children that participated in this study were referred to obesity center Heideheuvel by their pediatricians. Since not every severely obese child or adolescent in the Netherlands will have been under the care of a pediatrician and therefore was not able to participate in the study, participants of HELIOS were probably a selection from the total group of severely obese children and adolescents in the Netherlands. It is unknown if the children and adolescents not being referred by pediatricians differ in characteristics from the children and adolescents that were being referred. It is possible that the children and adolescents that were to seek help from a pediatrician were also the ones already suffering from cardiometabolic risk factors. Also, the inclusion of participants with a SDS-BMI between 2.3 and 3.0 with co-morbidity can lead to a preselected sample and therefore has a higher prevalence of these factors than a general population of obese children. In practice, 7 participants had a SDS-BMI between 2.3-3.0 (2.9, 2.8, 2.9, 2.5, 2.8, 2.6, and 2.5). Of these participants, 0 had impaired fasting glucose, 0 had impaired glucose tolerance, 0 had diabetes mellitus type II, 4 had insulin resistance, 1 had hypertension, and 6 had low HDL-cholesterol. Indeed, some of them already had cardiometabolic risk factors, but in general we saw a higher prevalence of these risk factors among those with a higher SDS-BMI. A strong point of this study is the large number of participants that came from all parts of the Netherlands. Many studies with a severely obese children and/or adolescents study population had fewer participants and participants for a limited area that did not present the country. This article was written since the prevalence of severe obesity is rising in children and adolescents worldwide; a good description of particularly this population is needed. The results in this article clearly demonstrate that most severely obese children and adolescents already have several cardiometabolic risk factors present and are at high risk for developing cardiometabolic disease in young adulthood. These findings stress the importance and need of the early detection of these children, of the availability of appropriate intensive treatment programs and of the early screening for cardiometabolic risk factors. It is even more important to prevent them from becoming severely obese, because effects of treatment can be lower than desired resulting from the complexity within the group of severely obese children and adolescents to prevent the development of cardiometabolic risk factors and diabetes and impaired quality of life.

## Conclusions

Cardiometabolic risk factors are already highly prevalent in this group of severely obese children and adolescents, 80% had at least 1 cardiometabolic risk factor (in addition to obesity). The score of 69.1 found for quality of life in this study suggests that participants experience important limitations in their quality of life. However, quality of life is not associated with the prevalence of cardiometabolic risk factors.

## Ethical and legal prerequisites

Trial registration: Netherlands Trial Register (NTR): NTR1678.

The Medical Ethics committee (METc) of VU University Medical Centre approved the study design, protocol and informed consent procedures. Written informed consents were obtained from both the participants and their parents.

## Competing interests

OHBS is affiliated with the treatment program as pediatrician. Other than that the authors declare that they have no competing interests.

## Authors’ contributions

All authors made substantial contributions to the conception and design of the study. SM, CMR and JEB drafted the manuscript. All authors were involved in revising the manuscript critically for important intellectual content. All authors read and approved the final manuscript.

## Pre-publication history

The pre-publication history for this paper can be accessed here:

http://www.biomedcentral.com/1471-2431/13/62/prepub

## Supplementary Material

Additional file 1 Definition of cardiometabolic risk factors. Click here for file
