# Supplementary material for: Cardiometabolic risk factors and quality of life in severely obese children and adolescents in the Netherlands
Source: BMC Pediatr. 2013 Apr 22;13:62. doi: 10.1186/1471-2431-13-62 (PMC3639189; doi:10.1186/1471-2431-13-62)
Supplement: Additional file 1 — Definition of cardiometabolic risk factors. [file 1471-2431-13-62-S1.doc]

Definition of cardiometabolic risk factors

|  | Children <16 | Children >=16 |  |
| --- | --- | --- | --- |
|  | Boys and Girls | Boys | Girls |
| Obesity | >= 90^th^ percentile (6- < 10) or >= 90^th^ percentile or adult cut-off if lower (10- < 16) | WC >= 94 cm for males | WC >= 80 cm for females |
| High triglycerides | >= 1.7 mmol/L or >= 150 mg/dL | >= 1.7 mmol/L or >= 150 mg/dL | >= 1.7 mmol/L or >= 150 mg/dL |
| Low HDL-cholesterol | < 1.03 mmol/L or < 40 mg/dL | < 1.03 mmol/L or < 40 mg/dL | < 1.29 mmol/L or < 50 mg/dL |
| Hypertension | Systolic BP >= 130 or diastolic BP >= 85 mm Hg | Systolic BP >= 130 or diastolic BP >= 85 mm Hg | Systolic BP >= 130 or diastolic BP >= 85 mm Hg |
| FPG or DMII | FPG >= 5.6 mmol/L (100 mg/dL)** or known DMII | FPG >= 5.6 mmol/L (100 mg/dL)** or known DMII | FPG >= 5.6 mmol/L (100 mg/dL)** or known DMII |

HDL – high-density lipoprotein; BP – blood pressure; FPG – fasting plasma glucose; DMII – type 2 diabetes mellitus.

Reference cut off points cardiometabolic risk from ‘The metabolic syndrome in children and adolescents – an IDF consensus report’ by Zimmet et al. [27].
